# Elevated pulmonary artery pressure and brain natriuretic peptide in high altitude pulmonary edema susceptible non-mountaineers

**DOI:** 10.1038/srep21357

**Published:** 2016-02-19

**Authors:** Rajinder K. Gupta, G. Himashree, Krishan Singh, Poonam Soree, Koundinya Desiraju, Anurag Agrawal, Dishari Ghosh, Deepak Dass, Prassana K. Reddy, Usha Panjwani, Shashi Bala Singh

**Affiliations:** 1 Defence Institute of Physiology and Allied Sciences. Timarpur, Delhi-110054, India; 2 CSIR Institute of Genomics and Integrated Biology, Mall Road, Delhi 110007, India

## Abstract

Exaggerated pulmonary pressor response to hypoxia is a pathgonomic feature observed in high altitude pulmonary edema (HAPE) susceptible mountaineers. It was investigated whether measurement of basal pulmonary artery pressure (Ppa) and brain natriuretic peptide (BNP) could improve identification of HAPE susceptible subjects in a non-mountaineer population. We studied BNP levels, baseline hemodynamics and the response to hypoxia (FIo_2_ = 0.12 for 30 min duration at sea level) in 11 HAPE resistant (no past history of HAPE, Control) and 11 HAPE susceptible (past history of HAPE, HAPE-S) subjects. Baseline Ppa (19.31 ± 3.63 vs 15.68 ± 2.79 mm Hg, p < 0.05) and plasma BNP levels (52.39 ± 32.9 vs 15.05 ± 9.6 pg/ml, p < 0.05) were high and stroke volume was less (p < 0.05) in HAPE-S subjects compared to control. Acute hypoxia produced an exaggerated increase in heart rate (p < 0.05), mean arterial pressure (p < 0.05) and Ppa (28.2 ± 5.8 vs 19.33 ± 3.74 mm Hg, p < 0.05) and fall in peripheral oxygen saturation (p < 0.05) in HAPE-S compared to control. Receiver operating characteristic (ROC) curves showed that Ppa response to acute hypoxia was the best variable to identify HAPE susceptibility (AUC 0.92) but BNP levels provided comparable information (AUC 0.85). BNP levels are easy to determine and may represent an important marker for the determination of HAPE susceptibility.

HAPE is hydrostatic edema which occurs in predisposed subjects at altitude above 2500 m^1^,^2^,^3^. The exact etiology of HAPE is not known, however, rapid ascent and individual susceptibility are important factors in the occurrence of HAPE^4^.

It is important to consider that HAPE represents an extreme pathological version of an otherwise physiological response to hypoxia, with various studies showing that exposure to chronic hypoxia enhances pulmonary artery pressures and vascular reactivity to acute hypoxia^5^,^6^. During ascent to high altitude there is a normal increase in pulmonary artery pressure that is initially reversible by oxygen administration, but HAPE susceptible (HAPE-S) subjects are prone to develop more severe pulmonary hypertension (PH) unresponsive to acute oxygen breathing^7^. It has been suggested that HAPE-S individuals may have residual structural changes in pulmonary vasculature that explain the fixed high pulmonary artery pressure^8^. It is hypothesized that there is a constitutional abnormality in HAPE-S individuals which causes such changes. Reduced vascular capacity, sub-clinical PH or ventricular dysfunction are such possibilities^9^,^10^,^11^,^12^. Previous studies on susceptibility to HAPE were done on mountaineers and experimental protocol involved rapid ascent to high altitude. It is well known that physical exertion associated with rapid ascent is an independent factor for occurrence of HAPE and this along with selection bias may account for negative findings regarding baseline differences in pulmonary vascular function^13^,^14^. In contrast, Indian armed forces personnel are routinely deployed at high altitude location in the Himalaya Mountains, without any previous mountaineering experience, but following a carefully controlled acclimatization schedule that lowers the risk of HAPE. Yet, despite these precautions during deployment, some of these personnel develop HAPE, presumably reflecting an underlying HAPE-S defect. Identification of a biomarker to screen HAPE-S subjects is a vital need in this context. In this study acute normobaric hypoxic stress was given to both HAPE-S and control subjects at sea level and their pulmonary and systemic hemodynamic responses were compared before and after hypoxia.

B type natriuretic peptide (BNP) is up regulated in pulmonary artery hypertension disease^15^. Studies in patients with pulmonary hypertension (PH) have demonstrated that plasma BNP levels are raised proportionally to the extent of right ventricular dysfunction, Ppa and PVR^16^. It is not known whether BNP may serve as a marker of sub-clinical pathology and HAPE susceptibility. Thus, an additional aim of this study was to examine whether HAPE-S subjects can be identified by elevated BNP levels.

## Methods

### Study population

We studied 11 Control and 11 HAPE-S who were categorized based upon their resistance or susceptibility to HAPE and were radiologically documented during their previous stay at high altitude. Our study population consists of Army troops who stayed at high altitude as a part of their tenure posting. All control subjects stayed at high altitude more than 2500 m for duration of 2 years of which 3 month stay was at altitude more than 4500 m. Both the groups were air inducted from sea level and had repeated exposures to high altitude. HAPE-S subject also stayed at high altitude more than 2500 m. The altitude of induction was 3500 m and everyone had to follow six day acclimatization schedule which includes complete rest for first two days followed by graded increase in physical activity for next four days of induction to high altitude. The HAPE-S participants suffered the illness during induction at 3500 m which was confirmed radiologically in spite of observing acclimatization schedule. All volunteers were nonsmoking natives of low altitude free of airway infection, and receiving no medication when data was collected. None of the subject had resided at high altitude within last 6 months before the baseline measurements were carried out in Delhi, India at an altitude 293 m above sea level. The group of HAPE-S subjects consisted of individuals with mean age, 31.91 ± 4.74 y; wt, 69.7 ± 6.29 Kg; height, 168.6 ± 6.24 cm who had developed at least one case of radiologically documented HAPE within last 5 years. Control subjects consisted of individuals with mean age, 29.82 ± 2.68 y; wt, 69.73 ± 7.88 Kg; height, 171 ± 3.38 cm who did not develop HAPE during their stay at high altitude. All experimental protocols were approved by Defence Institute of Physiology and Allied Sciences Ethics Committee for scientific experiments. Informed written consent was obtained from all participants before enrolment in the study. All methods were carried out in accordance with the approved guidelines and regulations.

### General Procedures

The subjects were investigated in the supine position while breathing synthetic gas mixtures consisting of 21 or 12% oxygen (FiO2 = 0.12) mixed in nitrogen. The hypoxic gas mixture corresponded to an altitude of 4500 m. Inhalation was performed via a tight fit face mask. Systolic blood pressure (SBP), Diastolic blood pressure (DBP), Mean blood pressure (MBP), heart rate (HR) and peripheral oxygen saturation (Spo_2_) were recorded before and at the end of 30 min. of hypoxic stress by using Multi parameter monitor, BPL, India.

### Determination of pulmonary artery systolic pressure^17^

Pulmonary artery hemodynamics was measured noninvasively using echocardiography. Echocardiography studies were performed with My Lab 30 Gold Line ultrasonograph (Esaote India). Standard parasternal and apical two dimensional views were obtained, and color flow directed pulse wave Doppler measurements of transvalvular flows and continuous wave Doppler measurements of tricuspid regurgitant flow were obtained. A single lead electrocardiogram was recorded on the ultrasonograph. Measures obtained using this noninvasive technique correlates closely with those obtained using cardiac catheterization. Pulmonary artery systolic pressure (sPpa) was calculated as follows


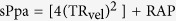


Where TR_vel_ is tricuspid regurgitation jet velocity and RAP is the estimated right atrial pressure based on respiration variation in inferior vena cava size.

Calculation of Mean pulmonary artery pressure^18^


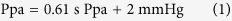


Cardiac output (Q) was calculated as follows


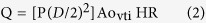


Where P(D/2)^2^ is the cross sectional area of blood flow into the aorta, Ao_vti_ is the velocity time integral through the aorta, and HR is the heart rate.

Systemic vascular resistance (SVR) was measured as follows^19^


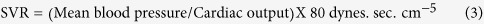


Pulmonary vascular resistance (PVR) was measured as follows^20^


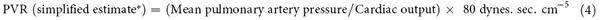


Measurements of sPpa, Q, SVR, PVR were obtained before and at the end of 30 min. of hypoxic stress.

*This is a simplified estimate, since the full expression is [(Mean pulmonary artery pressure–Pulmonary capillary wedge pressure)/Cardiac output] × 80 dynes. sec. cm^−5^.

### Pulmonary function measurement

Basal pulmonary function data was measured using dry, rolling seal spirometer (P.K. Morgan, Kent, UK). In addition to lung volumes and flow measurements, Functional residual capacity (FRC) was measured by the closed circuit Helium Dilution test. Diffusion capacity of the lung (DLCO) was also determined. The best value from three attempts were recorded as both absolute values and as percentage of the predicted values, based on age and body weight.

### Biochemical parameters

Baseline venous blood samples were collected in heparin tube, centrifuged and plasma stored at −80 °C until analyzed.

Plasma BNP levels (pg/ml) was measured by enzyme-immuno assay method (Elabscience; E-EL-H0598). The sensitivity of assay was 18.75 pg/ml. The coefficient of variation was <10%.

### Statistics

Corrected p values were used for multiple comparisons within and between the groups for normally distributed data. Non-parametric statistical tests were used for nonnormally distributed data. Wilcoxon signed rank test for paired and Wilcoxon rank sum test for comparison in between the two groups. ROC analysis was done for Ppa and BNP and cutoff values for all the variables, their sensitivities and specificities were calculated and compared. All the analysis was done using R statistical programming language.

All data are presented as means ± SD. P < 0.05 is considered significant.

## Results

### Baseline anthropometry and pulmonary function

The HAPE-S and control subjects were matched for age and lung function. Table 1 confirms that there were no significant differences in age, height and weight between two groups. There were no significant difference in forced vital capacity (FVC), Forced expiratory volume in 1 sec (FEV1), FEV1/FVC, Total lung capacity (TLC), Functional residual capacity (FRC), pulmonary diffusion capacity for carbon monoxide (DLCO) and Alveolar ventilation (VA), DLCO/VA between two groups.

### Hemodynamics before and after hypoxia

HAPE-S subjects had baseline higher Ppa and reduced stroke volume (SV) as compared to control. HAPE-S subject showed higher baseline levels of BNP compared to control. Hypoxia produced a significant fall in SVR (formula No. 3) in HAPE-S compared to control. An exaggerated increase in heart rate (HR), mean arterial pressure (MAP) and Ppa (formula No. 1) was seen in HAPE-S subjects in response to hypoxia compared to control. Hypoxia produced a fall in peripheral oxygen saturation (SpO_2_) in both the groups however HAPE-S showed exaggerated fall compared to control.

### Systemic and pulmonary hemodynamic parameters during normoxia and hypoxia

Heart rate (HR): There was no significant difference between two groups during normoxia. HAPE-S subjects showed an exaggerated increase in HR (p < 0.05) compared to control (23% in HAPE-S vs 15% in control) during hypoxia.

Blood pressure: There was no significant difference in systolic blood pressure (SBP), diastolic blood pressure (DBP) and MAP during normoxia. Hypoxia led to a significant increase in DBP and MAP in HAPE-S subjects, when compared to control.

Systemic vascular resistance (SVR): No significant difference in SVR was found during normoxia between the two groups. Hypoxia led to a significant fall in SVR in HAPE-S subjects, when compared to normoxia.

Cardiac output (Q): There was no significant difference in Q (formula No. 2) during normoxia between the two groups. Hypoxia led to a significant increase in cardiac output in HAPE-S (26% vs 14% in control).

Stroke volume (SV): HAPE-S subjects showed a significantly lower SV (24% less than control) during normoxia. No significant difference was observed during hypoxia between two groups.

Pulmonary vascular resistance (PVR): HAPE-S subjects showed a significantly higher PVR (31% greater than control) during normoxia. Hypoxia led to an exaggerated increase (p < 0.05) in PVR (formula No. 4) in HAPE-S (30% higher than control). All these findings are summarized in Table 2.

### Pulmonary artery pressure under normoxic and hypoxic condition and baseline brain natriuretic peptide levels in HAPE susceptible subjects

**Figure 1:**
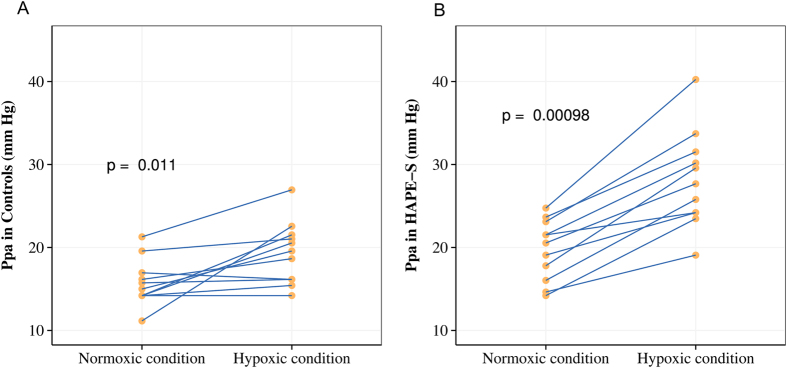
Mean pulmonary arterial pressure (Ppa) levels in normoxic and hypoxic conditions in controls (**A**) and in HAPE-S (**B**) individuals. To illustrate paired nature of data values of same individual are connected by a line. P values shown are obtained from paired t-test.

**Figure 2:**
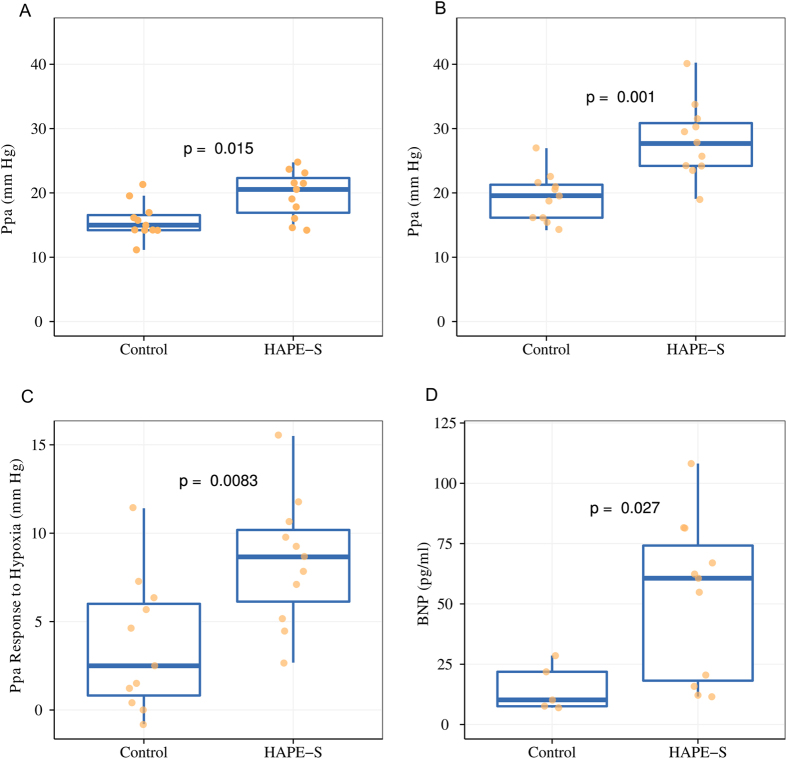
Boxplots showing comparison between control and HAPE-S individuals of mean pulmonary arterial pressure (Ppa) in normoxia (**A**) acute hypoxia (**B**), response to acute hypoxia (**C**) and baseline Brain natriuretic peptide (BNP) levels (**D**).

Figures 1 and 2 Pulmonary artery pressure (Ppa): HAPE-S subjects showed a significantly higher (p < 0.05) Ppa (25% higher than control) during normoxia. Hypoxia produced an exaggerated increase (p < 0.01) in Ppa in HAPE-S (43% vs 23% increase) compared to control.

HAPE-S subject showed significantly higher (p < 0.05) baseline levels of BNP (52.39 ± 32.9 vs 15.05 ± 9.6 pg/ml) compared to control. The value of ROC area under the curve (AUC) for basal BNP value was in the diagnostically suitable range and comparable to Ppa in hypoxic condition (0.85 vs. 0.92).

**Figure 3:**
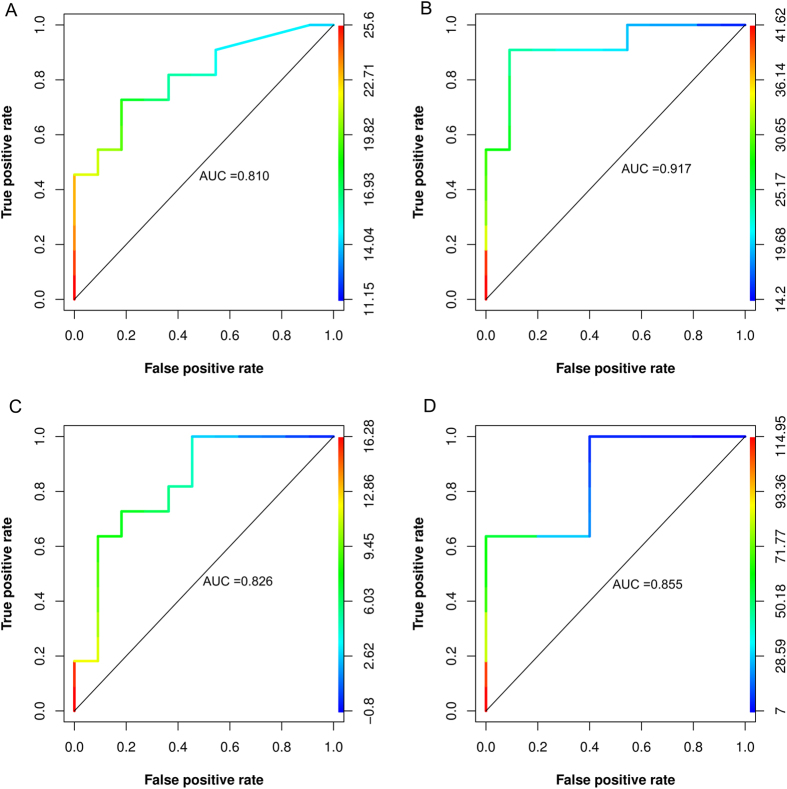
Receiver operator curves indicating the AUC for Pulmonary arterial pressure (Ppa) in normoxic (**A**) and hypoxic conditions (**B**), Ppa response to hypoxia (**C**) and baseline Brain natriuretic peptide (BNP) levels (**D**). Curves are color coded based on cutoff values which are shown as the second Y-axis.

ROC curves and values of area under the curve for pulmonary artery pressure under normoxic and hypoxic condition and baseline brain natriuretic peptide levels in HAPE-S subjects is summarized in (Fig. 3). The thresholds associated with points on the ROC curve can be read from the color coded secondary Y-axis.

## Discussion

Exaggerated increase in Ppa in response to acute hypoxia is a pathogonomic feature of HAPE susceptiblity in mountaineers and trackers^21^,^22^. In this study, we show that non-mountaineers who developed HAPE, have not only a similar exaggerated Ppa increase but also increased sub-clinical baseline Ppa. To the best of our knowledge, this is the first such study in, a non-mountaineer population that followed acclimatization schedule on arrival at high altitude. This increases the likelihood that HAPE-S subjects from this population are representative of a true HAPE susceptible population with underlying abnormalities that predispose to exaggerated responses to hypoxia. Additionally, our study shows for the first time that elevation of BNP is comparable to Ppa response to acute hypoxia in identifying subjects who are HAPE susceptible.

The major limitation of our study is that subjects were studied after the development of HAPE. In our soldier population that undergoes acclimatization, the incidence of HAPE is very low and prospective studies were not feasible. The possibility that the observed differences are a consequence of HAPE, rather than risk markers, appears unlikely for the following reasons. First, the possibility of residual effects of high altitude stay as a cause of PH at sea level in HAPE-S subjects can be ruled out since none of the subject had resided at high altitude within the preceding 6 months from baseline data collection. Second, DLCO/Va was not different between two groups. This shows that no persistent pulmonary membrane defect existed in subjects with previous history of HAPE. Similar results were shown in a larger set of subjects studied earlier^23^. Further, the well known noninflammatory nature of HAPE and its quick resolution with descent and/or oxygen speaks against residual traces of previous episode^24^. While aging itself causes an increase in Ppa^9^, subjects with lower age were included in the present study compared to earlier studies^14^. Other limitations include absence of pulmonary capillary wedge pressure measurements for more precise estimation of PVR, a short duration of hypoxia (30 min at 12% Fio_2_) and measurement of BNP at baseline but not post-hypoxia. In this group of non-mountaineers, 30 minutes of hypoxia led to substantial declines in oxygenation, as shown in Table 2. Further, our intent was to identify the utility of BNP as a rapid screening biomarker for HAPE susceptibility in our non-mountaineer population. Therefore, it was measured only at baseline. However, additional information provided by post-hypoxia measurement could have been useful for biological understanding.

Our findings are generally comparable to other reports, but with some important differences. Control subjects had resting normoxic Ppa and showed hypoxic pulmonary vascular response (HPVR) to acute normobaric hypoxia (FIO_2_ = 0.12) similar to that reported previously^21^,^25^,^26^. We found higher basal Ppa and PVR in HAPE-S subjects compared to control. This is in contrast to studies on mountaineers, which showed no difference in basal Ppa between two groups^13^,^14^,^24^,^26^. Studies showed that hypoxic pulmonary vasoconstriction caused by high altitude is comparable to breathing 4 hr of 12% oxygen at sea level. Normobaric hypoxia (12% oxygen) when given for 4 hr in mountaineer population showed Spo_2_ falls to 82 ± 2% in HAPE-S and 87 ± 2% in control^13^. In our population, Spo_2_ fell to 68 ± 11.8% in HAPE-S and 78.7 ± 3.8% in control after 30 min. of 12% hypoxia. Therefore, a strictly comparable frame of reference may not be available. Exposure to acute hypoxia resulted in abnormally high HPVR in HAPE-S which is in agreement with reports of other investigators^21^,^22^. Some investigators have not reported exaggerated HPVR to hypoxia^27^,^28^. The reason for this difference in HPVR is not clear but may be related to inter-individual variations in HPVR and HAPE susceptibility, different experimental design and different pathology like reentry HAPE^8^. Greater desaturation in HAPE-S subjects in the present study could be explained based on blunted ventilatory response to hypoxia, as has been reported in some studies, leading to lower levels of alveolar Po_2_^23^. However, reduced ventilatory response is an inconsistent observation and HAPE can occur in susceptible subjects despite the presence of a normal or high ventilatory response to hypoxia^29^.

Genetic variations in EGLN1 (HIF-prolyl hydroxylase 2), which normally hydroxylates HIF1α and marks it for degradation during normoxia, have been associated with HAPE^30^. EGLN1 expression was upregulated in larger set of HAPE patients (n = 250) and inversely correlated to SpO_2_ levels. Chronic hypoxia is closely associated with reduced expression of endothelial nitric oxide synthase which may explain low nitric oxide levels in pulmonary and systemic vasculature as also previously observed in HAPE-S^13^,^31^,^32^,^33^. Chronic hypoxia is known to cause vascular remodeling which explains baseline elevated Ppa and exaggerated pulmonary pressor response to acute hypoxia in the present study. Fall in SVR in response to acute hypoxia in HAPE-S is an exaggeration of a normal response since healthy individuals also show such hemodynamic response with increased severity and duration of hypoxia^34^.

Several reports have documented that HAPE resistant subjects have roughly 10% greater vital capacity that might indicate a smaller vascular bed in subjects susceptible to HAPE^8^,^12^. HAPE-R subjects have shown higher vascular capacity (Q and SV) during exercise compared to HAPE-S^12^. Since there was no ventilation perfusion mismatch at rest in between two groups therefore it was suggested that reduced vascular capacity could be a factor contributing to HAPE susceptibility^35^,^36^,^37^. Normal DLCO values in our HAPE-S subjects imply absence of any clinically significant reductions in baseline vascular capacity, but the possibility of sub-clinical differences that manifest during hypoxic stress cannot be excluded.

Dyspnoea on exertion (DOE) is a common symptom in both HAPE-S and pulmonary hypertension (PH) and associated with simultaneous rise in Ppa and pulmonary capillary wedge pressure (PCWP)^8^,^38^. Reduction of high PCWP in PH caused significant relief in DOE^38^. Acute hypoxia in HAPE-S induces increase in Ppa, as well as a possible rise in pulmonary capillary wedge pressure due to pulmonary venoconstriction in accordance to previous study^26^,^39^. This may cause pulmonary vascular congestion and arterial desaturation consistent with exaggerated fall in Spo_2_ in HAPE-S subjects in the present and past study^20^.

In the present study we have also found that the plasma BNP levels in HAPE susceptible subjects with no pulmonary disease were higher than healthy control subjects. It has been reported that an increase in BNP concentration is a risk factor for death independent of chronic lung disease^40^. These results were unchanged when factors related to age and gender were eliminated. Patients with HAPE showed increase in pulmonary artery pressure with normal left atrial pressures^41^. Pulmonary hypertension leading to shift of interventricular septum towards left was observed in 5 of 9 subjects with HAPE within first 24 hr of ascent to 4559 m^42^. BNP is transcribed in cardiac myocytes during mechanical strain, which supports our interpretation of the observed Ppa elevation being clinically relevant. Assuming that the observed increase in Ppa and BNP are related, as discussed above, it is useful to briefly consider the molecular pathways that intersect between PH and HAPE-S, since they would best describe the observed phenotype in our study like low levels of nitric oxide in pulmonary and systemic vasculature have been noted in both PH and HAPE-S^13^,^31^,^43^. Hypoxic response signaling has also been directly or indirectly related to both HAPE and PH and there is a strong rationale to further investigate variations in hypoxic response as a cause of the observed HAPE-S phenotype. The role of mitochondria in this context is particularly intriguing in view of their important role in oxygen sensing and free radical generation as mitochondrial redox distress has an association with HAPE susceptibility^44^.

Pulmonary hypertensive response to hypoxia and exercise has been found in relatives of patient with idiopathic and familial pulmonary artery hypertension^45^. The present study is first to associate elevated Ppa and baseline elevation of BNP with HAPE susceptibility. The potential use of BNP as a marker for HAPE susceptibility is attractive because of ease of testing. However, this is a very small dataset and larger independent studies are necessary before BNP can be considered to be a feasible screening approach, using consistent well-defined thresholds.

## Additional Information

**How to cite this article**: Gupta, R. K. *et al.* Elevated pulmonary artery pressure and brain natriuretic peptide in high altitude pulmonary edema susceptible non-mountaineers. *Sci. Rep.* **6**, 21357; doi: 10.1038/srep21357 (2016).

## Figures and Tables

**Table 1:** Baseline anthropometry and pulmonary functions in Controls and HAPE susceptible (HAPE-S) subjects.

	Control	HAPE-S	P Value
Age (yrs)	29.82 ± 2.68	31.91 ± 4.74	0.09
HT (cm)	171 ± 3.38	168.8 ± 6.24	0.26
WT (Kg)	69.73 ± 7.88	69.7 ± 6.29	1.00
FVC (Lt)	4.55 ± 0.39	4.33 ± 0.46	0.24
FVC%	94 ± 8.33	96.27 ± 13.84	0.65
FEV_1_ (Lt)	3.82 ± 0.34	3.46 ± 0.51	0.07
TLC%	100.09 ± 10.10	105.5 ± 17.03	0.96
FRC (Lt)	3.44 ± 0.58	3.29 ± 0.57	0.53
FRC%	112.2 ± 19.47	110.6 ± 20.08	0.85
DLCO (ml/kg/mm Hg)	37.28 ± 6.31	34.07 ± 6.02	0.24
DLCO%	114.3 ± 19.3	109.55 ± 20.82	0.58
VA%	75.9 ± 7.35	75.91 ± 7.05	1.00
DLCO/VA	124.3 ± 14.58	119.8 ± 16.4	0.50

Values are presented as Mean ± SD. FVC: forced vital capacity; FEV_1_: Forced expiratory volume in 1 sec; TLC: Total lung capacity; FRC: Functional residual capacity; DLCO: pulmonary diffusion capacity for carbon monoxide; VA: alveolar ventilation.

**Table 2:** Hemodynamic response to acute hypoxia in Control and HAPE–S subjects.

	Normoxia		Hypoxia	
	Control	HAPE–S	Control	HAPE–S
HR (beats/min)	60.91 ± 6.17	66.9 ± 7.42	70.45 ± 9.63*	82.5 ± 10.5*^a2^
SBP (mm Hg)	120.27 ± 11.9	122.73 ± 8.91	119.9 ± 8.92	127.4 ± 10.98
DBP (mm Hg)	67.18 ± 3.82	70.82 ± 6.01	66.45 ± 6.22	74.5 ± 6.38^a2^
MAP (mm Hg)	84.88 ± 6.19	88.76 ± 5.99	84.27 ± 5.78	92.1 ± 8.72^a2^
sPpa (mm Hg)	22.45 ± 4.58	29.04 ± 5.99^a1^	28.43 ± 6.13*	42.9 ± 9.5*^a2^
Ppa (mm Hg)	15.68 ± 2.79	19.71 ± 3.63^a1^	19.33 ± 3.74*	28.2 ± 5.8^*a2^
Q (Lt/min)	4.0 ± 0.54	3.72 ± 0.71	4.57 ± 1.33	4.7 ± 0.76*
Spo_2_ (%)	98.45 ± 0.82	98.18 ± 1.47	78.73 ± 3.8*	68.1 ± 11.8*^a2^
SV (ml)	68.08 ± 7.73	54.75 ± 10.42^a1^	64.21 ± 12.8	54.5 ± 6.9
SVR (dyne.sec.cm^−5^)	1723.96 ± 256.53	1953.11 ± 360.71	1586.3 ± 436.04	1622.4 ± 355.69*
PVR (dyne.sec.cm^−5^)	318.9 ± 72.2	419.01 ± 88.52^a1^	366.02 ± 125.71	477.7 ± 85.05^*a2^

Values are presented as Mean ± SD. HR: heart rate; SBP: systolic blood pressure; DBP: diastolic blood pressure; MAP: mean arterial pressure; sPpa: pulmonary artery systolic pressure; Ppa: mean pulmonary artery pressure; Q: cardiac output; Spo_2_: Peripheral oxygen saturation; SV: stroke volume; SVR: systemic vascular resistance; PVR: pulmonary vascular resistance.

a1: p < 0.05 (control versus HAPE susceptible under normoxia).

a2: p < 0.05 (control versus HAPE susceptible under hypoxia).

*p < 0.05 (normoxia versus hypoxia).
